# Comparison of the intraarticular osteotomy and the “window” osteotomy in the treatment of tibial plateau fracture involving depressed posterolateral fragments

**DOI:** 10.1186/s12891-023-06803-1

**Published:** 2023-08-30

**Authors:** Zhixun Fang, Xuan Pei, Yipeng Cheng, Jianan Chen, Wei Zhou, Yu Chen, Yaolatu Baosu, Shenglong Qian, Ximing Liu, Guodong Wang

**Affiliations:** 1 Department of Orthopedics, General Hospital of Central Theater Command, 627 Wuluo Road, Wuchang District, Wuhan City, Hubei Province China; 2 The First School of Clinical Medicine, Southern Medical University, Guangzhou City, Guangdong Province China; 3 School of Medicine, Wuhan University of Science and Technology, 2 Huangjiahuxi Road, Hongshan District, Wuhan City, Hubei Province China; 4 Hubei University of Chinese Medicine, 16 Huangjiahu West Road, Hongshan District, Wuhan City, Hubei Province China

**Keywords:** Tibial plateau fracture, Fracture reduction and fixation, Anterolateral supra-fibular-head approach, Intraarticular osteotomy, “Window” technique

## Abstract

**Objectives:**

The methods of reduction of depressed posterolateral fragments in tibial plateau fracture through anterolateral approaches remain controversial. This paper aimed to compare the intraarticular osteotomy technique and the “window” osteotomy technique for the reduction of depressed posterolateral fragments through anterolateral approach.

**Method:**

From January 2015 to January 2022, we retrospectively reviewed the data on patients with tibial plateau fracture involving depressed posterolateral fragments treated with the intraarticular osteotomy or the “window” osteotomy. 40 patients underwent the intraarticular osteotomy were divided into group A, while 36 patients underwent the “window” osteotomy were divided into group B. The operative time, bone grafting volume, fracture healing time, complication, reduction quality and postoperative functional results were compared between the two groups.

**Results:**

The average follow-up duration was 16.6 ± 3.7 months. The average bone grafting volume for all patients in group B was essential larger than group A (p = 0.001). Compared to group B, patients in groups A had significantly shorter fracture healing time (p = 0.011). The depth of depressed articular surface, PSA and the radiographic evaluation at 2 days and 6 months after surgery in group A were significantly lower than group B (p<0.05). Based on the HSS knee-rating score, no significant difference in function results was found between the two groups (p>0.05). No significant difference was found in operation time and blood loss between the two groups (p>0.05).

**Conclusion:**

The intraarticular osteotomy could obtain satisfactory clinical results in tibial plateau fracture involving posterolateral fragments.

## Introduction

Tibial plateau fractures have tended to be more common with the increased frequency of traffic accidents and are mostly caused by a combination of axial forces and varus or valgus to the knee [1–3]. Tibial plateau fracture involving posterolateral column account for 44.2% of tibial plateau fractures, which occur frequently in people over 50 years of age [4, 5]. The prognosis of patients with tibial plateau fracture depends on the articular reductions, even a minimal mal-reduction could be the reason of poor alignment of the lower limb force line and joint instability, which can lead to postoperative arthritis and total knee arthroplasty in the long term [6]. It was reported that articular mal-reductions of 2 mm or more may be related to the above consequence [7]. Hence, open reduction internal fixation is the optimal treatment for displaced tibial plateau fracture. However, due to the obstruction of the fibular head, the fibular collateral ligament (FCL) and posterolateral corner (PLC), and the various vessels and nerves (the popliteal artery/vein, the tibial nerve, the common peroneal nerve, etc.) surrounding the posterolateral column of the tibial plateau, it is difficult to obtain adequate vision of posterolateral fragments and thus flatten reduce the articular surface.

Currently, scholars have been dedicated to increasing the exposure of posterolateral articular surface of tibial plateau through various surgical approaches to facilitate direct visualization. However, there is still no optimal solution for the surgical treatment of tibial plateau fracture involving posterolateral fragments. The anterolateral supra-fibular-head approach proposed by Hu [8, 9] allows for direct visualization of posterolateral plateau through a space between the FCL and the lateral condyle and avoided the risk of nerves and vessels injury. However, it is difficult to reduce the posterolateral fragments through the anterolateral approach due to the obstruction of anterolateral cortex, especially for depressed posterolateral fragments. Due to the inability to directly access the posterolateral fragments, intraarticular osteotomy and “window” osteotomy becomes conventional method for reduction. However, there are few clinical studies comparing intraarticular osteotomy with “window” osteotomy in the treatment of tibial plateau fracture involving depressed posterolateral fragments [10].

This study aims to compare the intraarticular osteotomy and “window” osteotomy in the treatment of tibial plateau fracture involving depressed posterolateral fragments. We hope that this study will provide guidance for clinical practice in this type of fracture.

## Materials and methods

### Patients

From January 2015 to January 2022, we retrospectively reviewed the data on patients with tibial plateau fracture involving depressed posterolateral fragments treated with the intraarticular osteotomy or the “window” osteotomy at our hospital. The Institutional Research Ethics Committee approved the study, and all participants provided written informed consent.

The inclusion criteria included (1) tibial plateau fractures involving depressed posterolateral fragments; (2) posterolateral fragments depressed and displaced beyond 3 mm [11]; (3) age equal to or greater than 18 years. The exclusion criteria included (1) open tibial plateau fracture; (2) combined with vascular or never injury; (3) >3 weeks between the injury and the initial operation; (4) had incomplete patient datasets or were lost prior to the minimum study follow-up of one year.

**Fig. 1:**
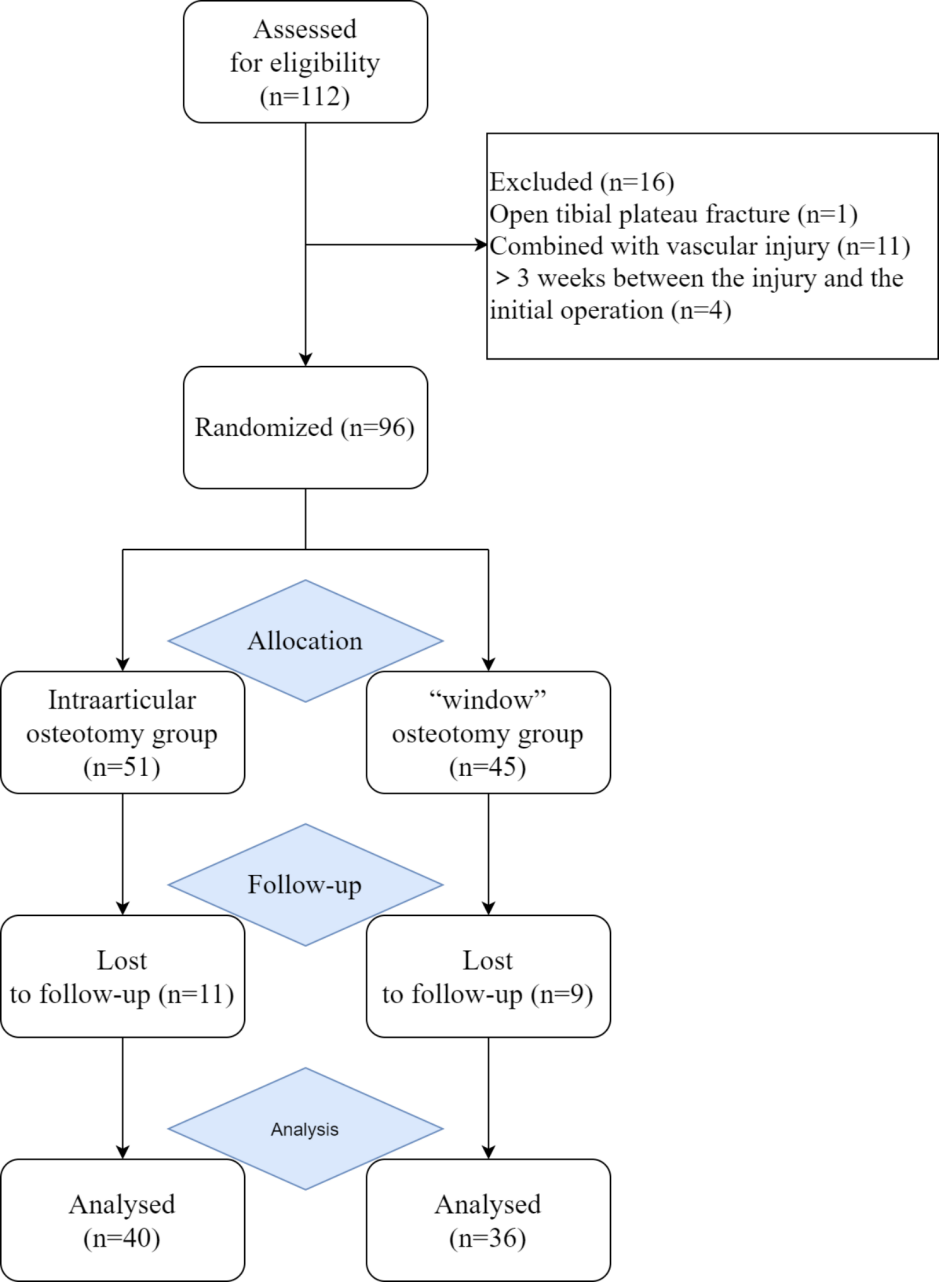
Patient flow chart

76 patients with tibial plateau fracture involving depressed posterolateral fragments were included in the study (Fig. 1). Patients were classified into group A and group B based on whether the surgery was carried with an intraarticular osteotomy or a “window” osteotomy. 40 patients underwent the intraarticular osteotomy were divided into group A, while 36 patients underwent the “window” osteotomy were divided into group B. Patient characteristics are summarized in Table 1, showing the comparability of demographics between both groups.

**Table 1:** Baseline characteristics of patients

Variables	Group A(n = 40)	Group B(n = 36)	Test value	P value
Age(year)	*44.0 ± 11.2*	*43.6 ± 14.7*	*t = 0.149*	*0.882*
Gender				
Male	*25*	*17*	*X*^*2*^ *= 1.789*	*0.181*
Female	*15*	*19*		
Fracture side				
Left	*25*	*23*	*X*^*2*^ *= 0.016*	*0.900*
Right	*15*	*13*		
Mechanism of injury				
Traffic accident	*11*	*10*	*X*^*2*^ *= 0.248*	*0.969*
Fall from height	*7*	*5*		
Fall from standing height	*15*	*15*		
Other injuries	*7*	*6*		
Schatzker classification				
II	*12*	*6*	*X*^*2*^ *= 4.556*	*0.207*
III	*1*	*5*		
V	*18*	*17*		
VI	*9*	*8*		
period between injury to surgery(days)	*6.4 ± 1.9*	*7.6 ± 4.0*	*t=-1.720*	*0.090*

### Preoperative planning

Routine preoperative examinations consisted of anteroposterior and lateral X-ray and CT scans (axial, 2D and 3D reconstruction) of knee joint. All the patients were given low molecular heparin sodium to prevent low limb deep venous thrombosis. All the patients were immobilized with casts, braces or calcaneal traction preoperatively. The surgery schedule was based on the condition of the soft tissue.

### Surgery procedure

All of procedures in this study were performed by the same surgical team, with the same lead surgeon. All patients were placed in the supine position on a radiolucent table. A tourniquet was placed on the thigh. The knee was put in modest flexion and mild adduction with surgical towels rolled into a cylinder placed slightly laterally under the knee. We used the anterolateral supra-fibular-head approach [8, 9] to expose the anterolateral tibial plateau. A 10-cm-long skin incision was performed from the Gerdy’s tubercle, extending proximally and posteriorly, crossing over the fibular head, ending 1 to 2 cm above the knee joint surface. The subcutaneous tissue was dissected to expose lateral plateau and iliotibial band (ITB). The ITB was incised partial alone the direction of the fibers, anterior tibial muscle group insertions were dissected anteriorly to allow for the placement of the plate. Then we incised the coronal ligament and joint capsule horizontally above the lateral plateau. After cleaning the hematoma in the joint, the lateral plateau was exposed. For a better surgical vison, the knee was put 60°flexion with slight adduction of the lower leg.

**Fig. 2:**
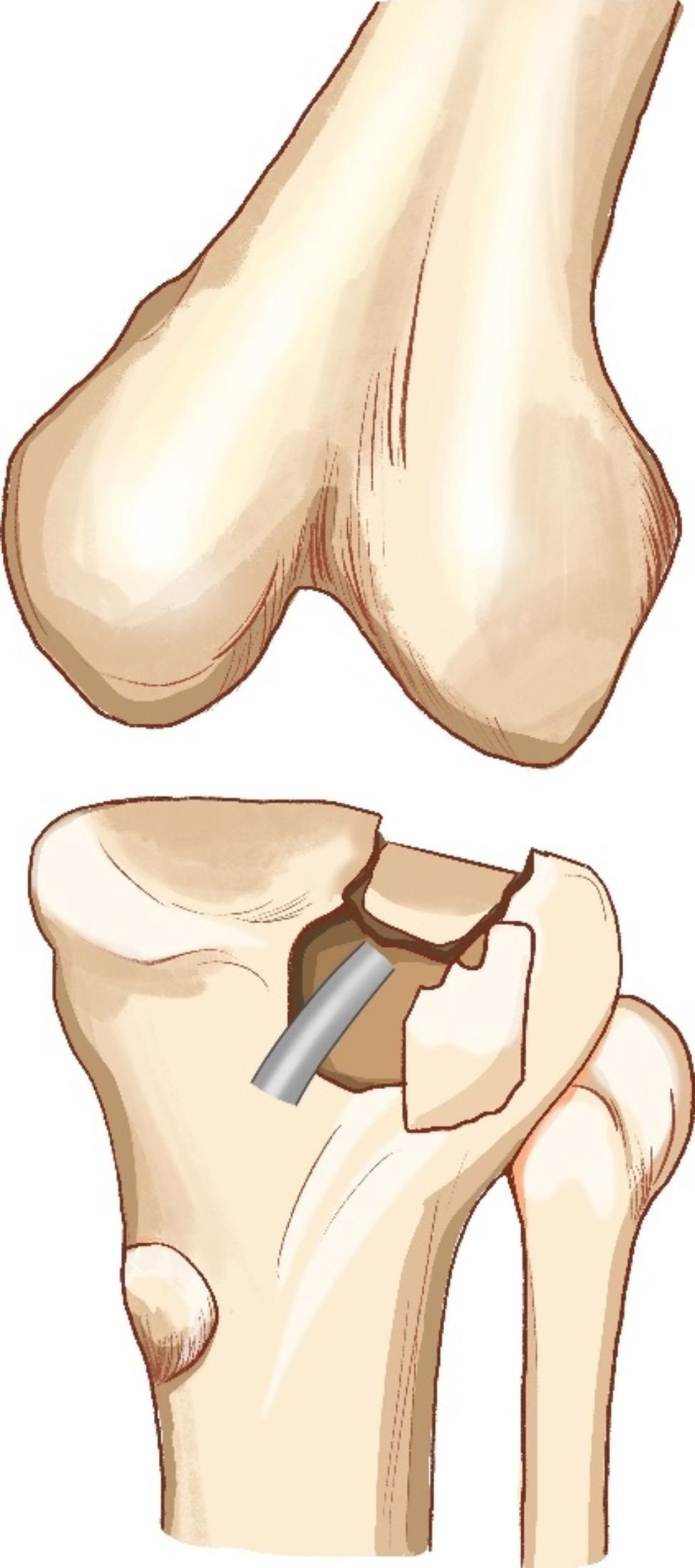
An illustrative diagram of the intraarticular osteotomy for tibial plateau fracture with posterolateral fragment

**Fig. 3:**
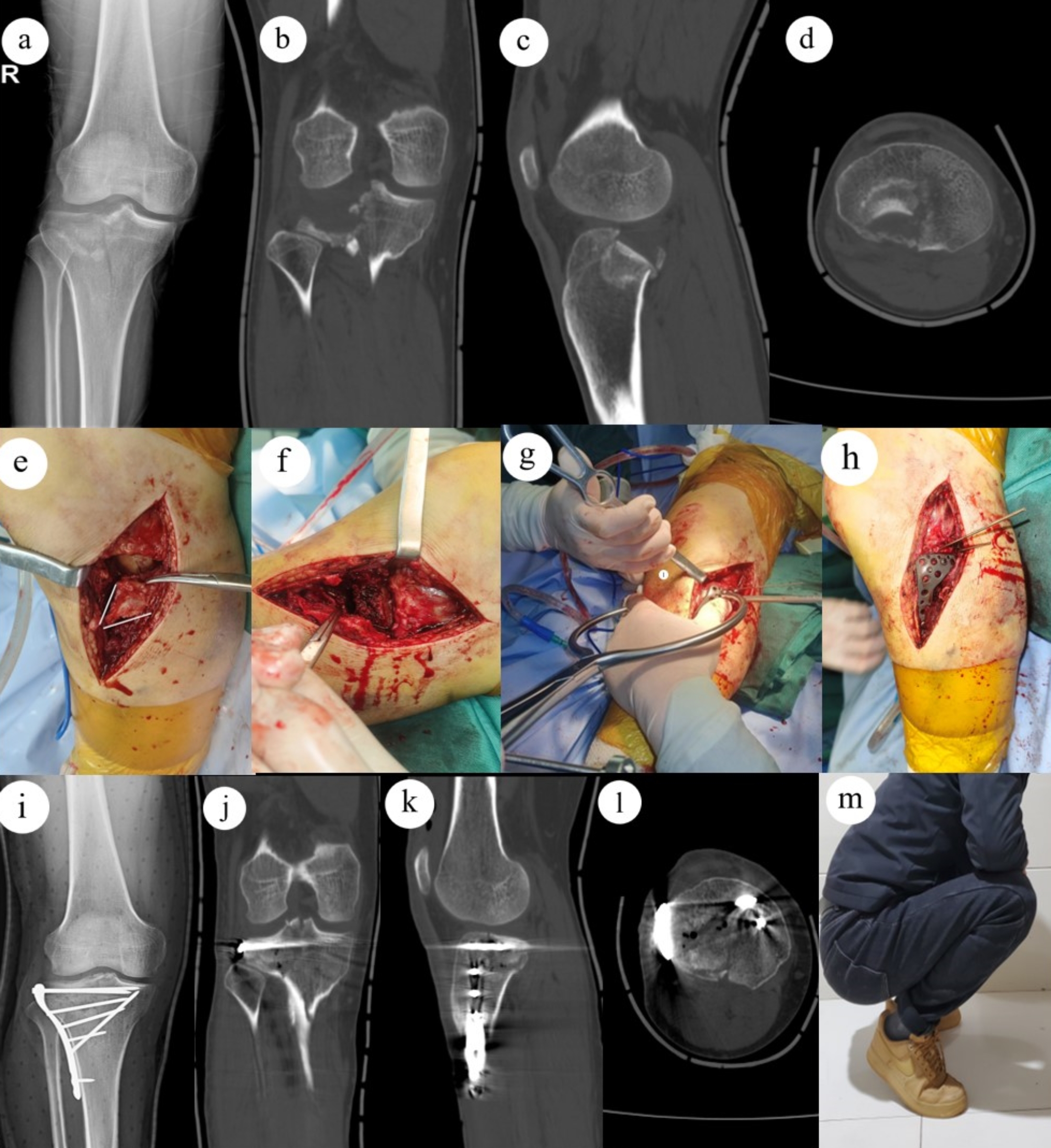
A case of type V tibial plateau fracture, male, 29 years old. **a-d** Preoperative X-ray and CT showed an obvious depressed posterolateral tibial plateau fracture and a splitting medial tibial plateau fracture. **e** The osteotomy lines are outlined by double white line. f direct visualization and manipulation of depressed posterolateral fragment were available with the external rotation of osteotomy fragment. g Intraoperative image showed the medial and lateral condyles were fixed temporarily with bone tenaculum to prevent widening of the plateau. h Intraoperative image showed a lateral locking plate was placed on the lateral rim of the tibial plateau. i-m Postoperative X-ray and CT showed that the fracture was anatomically reduced. **j** This patient’s 6-months follow-up

In group A, osteotomy was performed at the cortex of anterolateral plateau by a sharp osteotome. The vertical osteotomy line was designed to connect to the medial edge of the posterolateral fragments, while the horizontal osteotomy line should be 1.5 cm below the lowest point of the posterolateral fragments and extend laterally to allow for fragments elevation and plate displacement. After external rotating the anterior cortex, we got the access to posterolateral fragment under direct vision. A sharp osteotomy was used to elevate the depressed posterolateral fragment until the lateral plateau was level. The cavity was adequately filled with allogeneic bones. After reduction, the Kirschner wires were used for temporarily fixation. The medial and lateral condyles were fixed temporarily with bone tenaculum to prevent widening of the plateau. After determining the depressed fragment was satisfactorily reduced by C-arm, a lateral locking plate was placed on the lateral rim of the tibial plateau. A raft of four screws were screwed to fix the posterolateral fragments and osteotomy fragment, two for fixing the posterolateral fragment, and two for fixing the osteotomy. If the posterolateral fragment is too small to be fixed with two screws, one screw will be used for fixation, with a jail screw [11] or a Kirschner wire placed from anterior to posterior to reinforce fixation. The lateral meniscus and coronary ligament were sutured with 2 − 0 absorbable sutures. Repair of subcutaneous tissue and skin was performed as usual. An illustrative diagram of the intraarticular osteotomy for tibial plateau fracture with posterolateral fragment is shown in Fig. 2. A typical case is shown in Fig. 3.

**Fig. 4:**
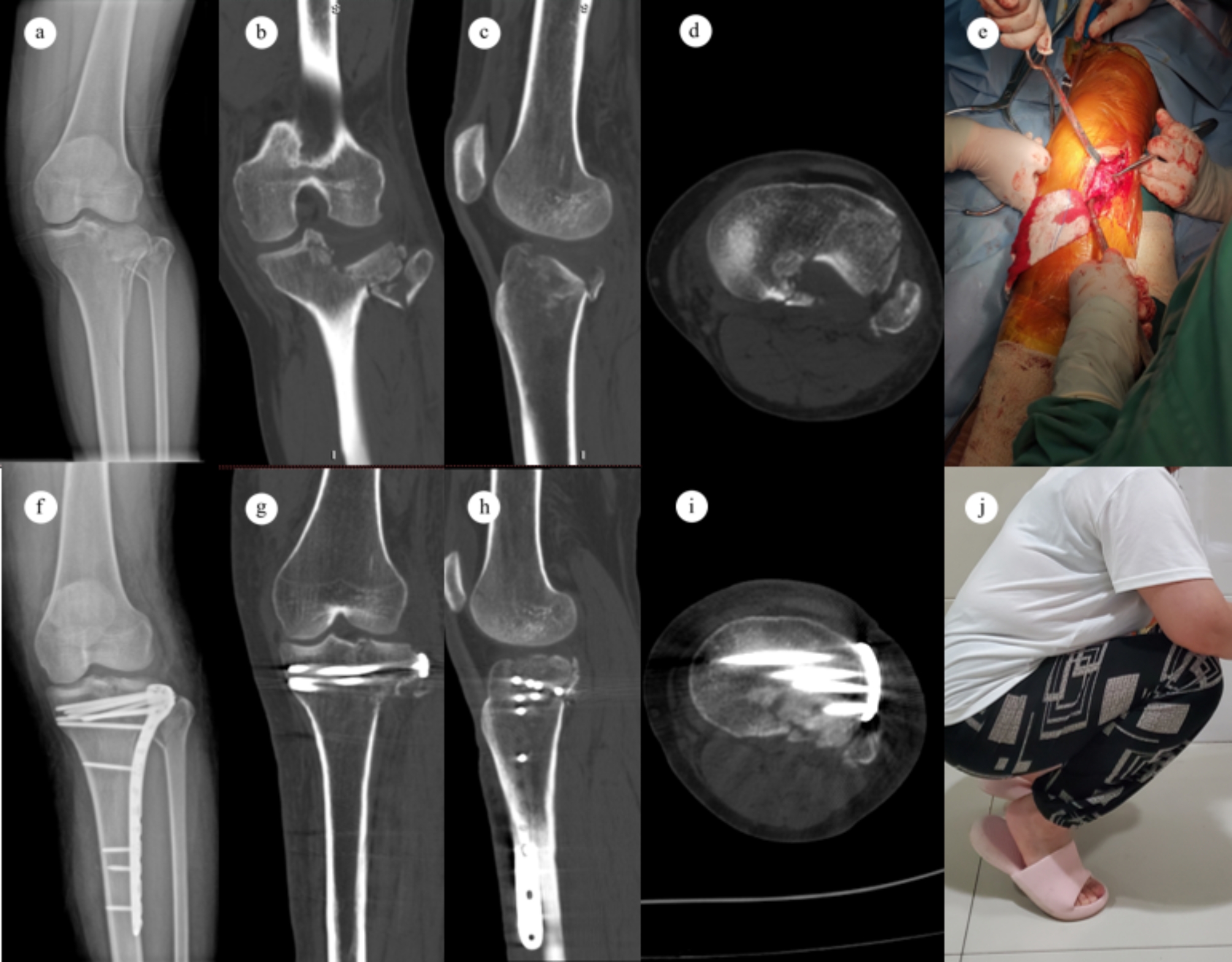
A case of type II tibial plateau fracture, female, 64 years old. **a-d** Preoperative X-ray and CT showed a significant depressed fragment in posterolateral plateau. **e** Intraoperative image showed a cortex window was created on the anterolateral plateau and the depressed posterolateral fragments were elevated through the window with a metal bone tamp. **f-i** Postoperative X-ray and CT showed that the fracture was anatomically reduced. **j** This patient’s 6-months follow-up

In group B, a cortical window was created 5 cm below the anterolateral articular surface. With the help of lateral articular surface exposure through the space between the FCL and the lateral condyle, the depressed posterolateral fragments were carefully elevated through the cortical window with a metal bone tamp until the lateral plateau was level. The cavity was adequately filled with allogeneic bones. The following implant fixation was performed as group A. The lateral meniscus and coronary ligament were sutured with 2 − 0 absorbable sutures. Repair of subcutaneous tissue and skin was performed as usual. A typical case is shown in Fig. 4.

### Postoperative Rehabilitation

All patients were treated with a standardized postoperative rehabilitation protocol. Knee range of motion (ROM) exercises not exceeding 90°was performed under the protection of adjustable knee brace after 3 days postoperatively and ROM exercises exceeding 90°can be performed after 6 weeks postoperatively. After the fracture had been radiographically confirmed to be stable enough, all the patients were guided to have standing exercise and walking exercise until they were fully weight-bearing.

### Result evaluation

We retrospectively reviewed the result of patients with tibial plateau fracture involving depressed posterolateral fragments treated with the intraarticular osteotomy or the “window” osteotomy at our hospital from January 2015 to January 2022. Operation time, blood loos, bone grafting volume, fracture healing time, and complication in both groups were recorded and compared.

We evaluated the reduction results of both groups using CT scans or anteroposterior and lateral X-ray preoperatively, 2 days and 6 months postoperatively. Radiographical results of both groups were assessed with Rasmussen scores [12] and posterior slope angle (PSA). Based on the criteria of Rasmussen [12] the quality of the tibial plateau was evaluated and scored radiographically, according to the joint surface depression, condylar widening and angulation (valgus/ varus), each of which is scored 6 points, with a total score of 18 points. The tibial plateau fracture reduction was expressed as excellent (18 points), good (12–17 points), fair (6–11 points) and poor (<6 points).

During the follow-up, hospital of special surgery (HSS) knee-rating score [13] was adopted for knee function evaluating. Based on the pain, walking and standing function, range of motion, muscle strength, flexion deformity and knee instability, all the patients were graded as excellent (≥ 85 points), good (70–84 points), fair (60–69 points) and poor (<60 points).

At each review, the healing and the fracture reduction loss of both groups were assessed and compared with CT scans or anteroposterior and lateral X-ray.

### Statistics

SPSS software version 26.0 was adopted for statistics. Test of normality was performed for variables in the means of Kolmogorov-Smirnov test. Variables with a normal distribution were expressed as $\bar{\text{x}}$± s. comparison of variables of both groups at different time points was performed using ANOVA. Variables that not follow normal distribution were expressed as M (Q_1_, Q_2_). Mann-Whitney U test and Kruskal-Wallis H test were used for these variables as appropriate. P value<0.05 was defined as statistical significance.

## Results

### Clinical data

The average follow-up duration was 16.6 ± 3.7 months (range, 12–28 months). Demographic data, fracture side, mechanism of injury, fracture classification and the period between injury to surgery are presented in Table 1. No significant difference was found in demographic data, fracture side, mechanism of injury and the period between injury to surgery between the two groups (p>0.05). The bone grafting volume in group B was larger than group A (3.8 ± 1.7 cm^3^ vs. 5.3 ± 2.1 cm^3^ for group A vs. group B, p = 0.001; Table 2). No significant difference was found in operation time and blood loss between the two groups (p>0.05; Table 2). The fracture healing time in group A was significantly lower than group B (13.1 ± 2.1 weeks vs. 14.4 ± 2.3 weeks, p = 0.011; Table 2).

**Table 2:** Surgical and clinical outcomes

*Variables*	*Group a(n = 40)*	*Group b(n = 36)*	*Test value*	*P value*
*Bone grafting volume (cm* ^*3*^ *)*	*3.8 ± 1.7*	*5.3 ± 2.1*	*t=-3.443*	*0.001*
*Operation time (minutes)*	*141.3 ± 28.6*	*153.4 ± 25.3*	*t=-1.942*	*0.056*
*Blood loss (ml)*	*119.5 ± 74.5*	*126.9 ± 54.8*	*t=-0.491*	*0.625*
*Fracture healing time (weeks)*	*13.1 ± 2.1*	*14.4 ± 2.3*	*t=-2.595*	*0.011*
*Depth of depressed articular surface (mm)*				
*Preoperative*	*13.9 ± 5.7*	*11.8 ± 5.6*	*t = 1.606*	*0.113*
*2 days*	*0.8 ± 0.2*	*1.0 ± 0.3*	*t=-4.082*	*> 0.001*
*6 months*	*0.9 ± 0.2*	*1.2 ± 0.3*	*t=-5.460*	*> 0.001*
*PSA (°)*				
*Preoperative*	*21.9 ± 3.0*	*21.8 ± 3.1*	*t=-0.210*	*0.835*
*2 days*	*9.4 ± 1.5*	*10.1 ± 0.9*	*t=-2.336*	*0.022*
*6 months*	*9.3 ± 1.5*	*10.2 ± 1.2*	*t=-2.698*	*0.009*
*Radiological evaluation* *(rasmussen classification)*				
*Preoperative*	*6.5 ± 1.7*	*6.4 ± 1.6*	*t = 0.270*	*0.788*
*2 days*	*16.5 ± 1.4*	*15.6 ± 1.6*	*t = 2.288*	*0.025*
*6 months*	*15.7 ± 1.2*	*14.3 ± 1.4*	*t = 4.290*	*> 0.001*
*Functional evaluation* *(hss classification)*				
*6 months*	*88.7 ± 4.8*	*86.5 ± 6.0*	*t = 1.764*	*0.082*
*1 year*	*93.1 ± 5.0*	*92.2 ± 5.6*	*t = 0.716*	*0.476*

### Radiographic evaluation

The preoperative, 2-day-postoperative and 6-month-postoperative depth of depressed articular surface and PSA of both groups are presented in Table 2. No significant difference was found in preoperative depth of depressed articular surface and PSA of both groups (p>0.05). The 2-day-postoperative and 6-month-postoperative depth of depressed articular surface and PSA in group A were significantly lower than group B (p<0.05). According to Rasmussen scoring system, the postoperative radiographic outcome in group A was markedly higher than group B (p<0.05).

### Functional evaluation

Based on the HSS knee-rating score, in group A, the function results at the 6-month-postoperative follow-up were graded as excellent in 30 cases (75%), good in ten cases (25%), while the function results, in group B, were graded as excellent in 26 cases (72.2%), good in ten cases (27.8%). No significant statistical difference was found in the function outcomes between both groups (p = 0.082; Table 2). Besides, at the one-year-postoperative follow-up, the function results in group A presented excellent in 37 cases (92.5%) and good in three cases (7.5%), and the outcome in group B was graded as excellent in 29 cases (80.5%), good in seven cases (19.5%). It represented no significant difference in long-term functional result between both groups(p = 0.476; Table 2).

### Complications

At the final follow-up, no fracture re-displacement, varus or valgus knee deformity, knee instability and posttraumatic arthritis were found in both groups. None of patients developed plate failure and screw loosening at the follow-up. Lower extremity venous thrombosis occurred in three patients, two of group A and one of group B. In group A, one patient developed fat liquefaction in the incision and resolved with dressing changes. Postoperative superficial incision infection occurred in one patient in both groups, respectively, which resolved with antibacterial treatment and dressing changes. Another patient presented joint stiffness due to lack of early functional exercise in group B. There was not statistically difference in complication rate between the two groups (4/40 vs. 3/36 for group A vs. B, p>0.05).

## Discussion

Posterolateral fragments are not rare in tibial plateau fracture and have been always a challenge for orthopedic surgeons [14, 15]. The main purpose of surgery is to obtain a knee with normal motion, good alignment, painlessness and stability to allow for rapid postoperative rehabilitation. The quality of intraoperative reduction has the greatest impact on postoperative outcomes of tibial plateau fractures, remaining joint steps of more than 2 mm after reduction could lead to poorer clinical results and higher rates of postoperative traumatic arthritis and total knee arthroplasty [16–18]. Hence, it is important to reduce the posterolateral fragments with anatomic reconstruction of the articular surface. However, the exposure and reduction of posterolateral fragments remain a relatively challenging task for orthopedic surgeons due to the obstruction of anterolateral cortex, fibular head and posterolateral corner complex [19].

Currently, there is no optimal treatment for posterolateral fragments of tibial plateau [19–21]. For the purpose of direct exposure of the posterolateral fragments and the placement of the buttress plate, which is the strongest fixation method for posterolateral shearing tibial fracture proven by biomechanical testing [22], some surgeons have used posterior approaches for the treatment of posterolateral fragments [5, 23–26]. Posterior approaches broadly included direct posterior approaches, posterolateral approaches and posteromedial approaches. Bhattacharyya et al. [23] designed a direct posterior approach by performing a S-shaped incision on the center of popliteal fossa and retracting media gastrocnemius. Posterolateral approaches proposed by Carlson et al. [24], Frosch et al. [25] and Tao et al. [26] made access to posterolateral plateau by performing posterolateral incision and retracting the gastrocnemius and the soleus. Luo et al. [5]has described a posteromedial approach using an inverted L-shaped incision on posteromedial aspect of the knee with medial head of the gastrocnemius retracted laterally. Although posterior approaches provided direct access to posterolateral fragments, the narrow and deep manipulation field caused by the thick gastrocnemius make it hard to reduce and fix. Besides, the risk of injury to the peroneal nerve, the sural nerve and the popliteal vessel is troubling. In addition, for depressed posterolateral fragments, especially those who do not involve the posterior cortex, posterior approaches are difficult to manage.

The authors prefer anterolateral supra-fibular-head approach proposed by Hu et al. [8, 9] for the treatment of posterolateral fragments. The advantages of this approach include:(i) direct visualization of the flatness of the articular surface after reduction is available; (ii) exposure of the entire lateral plateau allows for the placement of lateral locking plate;(iii) injury of nerves and vessels can be avoided.

However, direct access to the posterolateral fragments through the anterolateral supra-fibular-head approach is not available because of obstruction of the anterior bone cortex. Methods of manipulation and reduction for posterolateral fragments are controversial. Several researches [27–29] have described the “window” technique to access to posterolateral fragments and determined the clinical outcome. Surgeons [30–32] have designed various method of intraarticular osteotomy to achieve direct visualization and reduction of posterolateral fragments. Recently, inflatable bone tamp reduction for the treatment of depressed posterolateral fragments has gradually caught the eyes of researchers, which has the advantage of less invasion [33, 34]. However, there is a lack of clinical testing for inflatable bone tamp reduction. The union of bone defect is still in doubt for this method. The prevailing methods of reduction for posterolateral fragments are still “window” technique and intraarticular osteotomy.

The intraarticular osteotomy technique and the “window” osteotomy technique are the two main surgical methods applied in the reduction of posterolateral fragments. The main distinction between the two technique lies in the method of accessing posterolateral fragment. The intraarticular osteotomy technique accesses the posterolateral fragments by external rotation of the osteotomy fragment, while the “window” osteotomy technique does this by creating a cortical window on cortex of anterolateral plateau. Both techniques have their advantages and disadvantages. The intraarticular osteotomy can reduce under direct visualization of posterolateral fragments, while the “window” osteotomy technique elevates the depressed fragments through the anterior cortical window and observes the result after reduction through the space between the FCL and the lateral condyle, which may be the reason why the quality of radiographic results in intraarticular osteotomy group are better than the “window” osteotomy group. In terms of bone grafting volume, the intraarticular osteotomy group is lower than the “window” osteotomy group in our study. We considered the reason is that an impact force, in “window” osteotomy technique, is applied to the depressed posterolateral fragments by a metal bone tamp to flatten the articular surface, while it exacerbates the compression of posterolateral fragments, resulting an increase in bone grafting volume required. The intraarticular osteotomy, on the other hand, allows for careful elevation of depressed posterolateral fragments under direct visualization, without exacerbating the compression. Besides, due to the inability to reduce posterolateral fragments under direct visualization, it is hard for the “window” osteotomy technique to control the force applied to the depressed posterolateral fragments. Excessive force always leads to additional damage to the articular surface.

There are some concerns that damage to articular cartilage is unavoidable with intraarticular osteotomy, which may be related to unsatisfactory results. However, we consider it not to be of much concern because the intraarticular osteotomy is performed in a low-energy controlled fashion. In our study, the intraarticular osteotomy in our study was performed vertical to the articular surface, in which case the damage to the articular cartilage was limited. Besides, the vertical osteotomy technique helps the articular surface to achieve a complete anastomosis under direct visualization when the tibial plateau was reduced. According to further study [35–37], an uneven articular surface may lead to unsatisfactory results, a vertical osteotomy helps increase stability and bony union. In the procedure of intraarticular osteotomy, ensuring the anatomical reduction of articular surface and osteotomy fragment is of great significance. Although no patients with intraarticular osteotomy developed related knee complications in our study, the damage to articular cartilage is still a question worth exploring. Given the advances in technology, a 3D-printed osteotomy guide plate based on pre-operative CT imaging data may help to achieve accurate and safe osteotomy, further avoiding the damage to articular cartilage [38].

There are also some limitations in the present study. First, our study is a retrospective design with a relatively small sample size. Second, as a retrospective study, the inherent weakness and biases of such study designs are inevitable. Further prospective randomized controlled trials with a large sample size are needed.

## Conclusion

This study assessed the intraarticular osteotomy technique and the “window” osteotomy technique for the reduction of depressed posterolateral fragments of tibial plateau fracture. It seemed that compared with the “window” osteotomy technique, patients who were performed with the intraarticular osteotomy have better radiographic results, shorter fracture healing time from our cases.

## Data Availability

The datasets used or analyzed during the current study are available from the corresponding author on reasonable request.
